# Working with Respiratory Illness: Presenteeism Among Healthcare Personnel at Tertiary-Care Hospitals in Bangladesh, 2008–2016

**DOI:** 10.1017/ash.2021.149

**Published:** 2021-07-29

**Authors:** Syeda Mah-E-Muneer, Md. Zakiul Hassan, Mejbah Uddin Bhuiyan, Kamal Hussain, Zubair Akhtar, Mustafizur Rahman, A. Danielle Iuliano, Eduardo Azziz-Baumgartner, Fahmida Chowdhury

## Abstract

**Background:** Healthcare personnel (HCP) in crowded and resource-poor countries (eg Bangladesh), might be at risk of exposure to and transmission of respiratory illnesses to coworkers, patients, and caregivers. The infection control practices in public hospitals are inadequate in Bangladesh. We estimated the incidence of respiratory illness episodes among HCP, and proportion of HCP who worked during respiratory illnesses, including influenza virus infection, at 2 tertiary-care public hospitals in Bangladesh. **Methods:** From May 2008 to February 2016, HCP (defined as physicians, nurses, interns, patient care assistant, cleaners, and administrative staff working in adult and pediatric medicine wards) were asked to self-report to study physicians when they experienced new onset of cough, rhinorrhea, difficulty breathing, or fever during the April–September influenza epidemic period each year. Study physicians followed HCP throughout their respiratory illness episodes and recorded respiratory symptoms, onset dates, duration of illness, and days of presenteeism and absenteeism during illness. Nasopharyngeal and oropharyngeal swabs were collected after informed written consent and were tested for influenza by rRT-PCR. We used hospital records to enumerate total HCP working in the study wards during influenza season and multiplied by 6-months follow-up per year to calculate person-time contribution for estimating respiratory illness incidence. **Results:** HCP self-reported 107 episodes of respiratory illness during 656 person years of follow-up, for an estimated incidence of 16.3 per 100 person years (95% CI, 13–20). Of 107 episodes, 33 (31%) included fever and cough. The mean illness length was 3.9 days (SD, ±1.8). HCP worked an average of 3.4 days (SD, ±1.4) while ill. HCP missed work for a median of 1 day (IQR, 1–2) during 29 (27%) of 107 illness episodes. HCP consented to collect swabs during 56 (52%) episodes, and among them 8 (14%) of 56 tested positive for influenza (flu-A, n = 5; flu-B, n = 3). Also, 63% of HCP with influenza reported fever and cough. HCP experiencing either respiratory illness or influenza worked for similar periods of days while ill: mean, 4 (SD, ±2.2) versus mean, 3.3 (SD, ±1.4) (*P* = .257). HCP worked during 105 (98%) of 107 respiratory illness and 7 (88%) of 8 influenza episodes. **Conclusions:** Most HCP in Bangladesh, including those with influenza, worked during respiratory illnesses. The potential value of stay-at-home policies, compensation for sick days, and influenza vaccination in reducing HCP-associated respiratory pathogen transmission could be assessed in Bangladesh and similar settings.

**Funding:** No

**Disclosures:** None

